# Population genetics data help to guide the conservation of palm species with small population sizes and fragmented habitats in Madagascar

**DOI:** 10.7717/peerj.3248

**Published:** 2017-05-03

**Authors:** Lauren M. Gardiner, Mijoro Rakotoarinivo, Landy R. Rajaovelona, Colin Clubbe

**Affiliations:** 1 Conservation Science, Royal Botanic Gardens, Kew, Richmond, Surrey, United Kingdom; 2 Département de Biologie et Ecologie Végétales, Faculté des Sciences, Université d’Antananarivo, Antananarivo, Madagascar; 3 Kew Madagascar Conservation Centre, Ivandry, Antananarivo, Madagascar

**Keywords:** Arecaceae, Dypsis, Itremo, Madagascar, Palms, Population genetics, AFLP, Biodiversity hotspot, Management, Protected areas

## Abstract

**Background:**

The need to incorporate genetic data into conservation management decisions is increasingly recognised. However, many published studies represent a ‘gold standard’ of sampling, techniques, and analyses. Such rigour is often not possible with limited funding and resourcing available for developing plans for the increasing number of threatened species requiring conservation management. Two endemic palm species of the Itremo Massif in central Madagascar, *Dypsis ambositrae* and *D. decipiens*, are known to be threatened with extinction and conservation management for these species is a priority for the newly created protected area in the region.

**Methods:**

The genetic diversity of these two species was studied using the relatively low-cost and rapid AFLP technique. DNA fragments generated using three primer combinations were analysed for 20 and 50 individuals of the two species, respectively, from across their ranges.

**Results:**

Genetic diversity was relatively low for both species. The two sites where the highly restricted *D. ambositrae* grows were found to be genetically distinct (although overall heterozygosity was low). Despite having a much wider distribution and relatively large population, *D. decipiens* did not show clear geographical nor genetic groupings and had similarly low genetic heterozygosity to *D. ambositrae*.

**Discussion and Recommendations:**

With so few individuals remaining in the wild and two genetically distinct subpopulations, it is recommended that both sites of *D. ambositrae* are conserved and that seed are collected from both for *ex situ* conservation and potential future reintroduction. It may be less important to focus resources on conserving or collecting *ex situ* material from all sites where *D. decipiens* is found, as the genetic diversity represented by each subpopulation is limited and increasing sampling may not protect significantly higher levels of genetic diversity. This study provides data that inform and support conservation decisions taken for both species within this region, and in the management of the newly designated Itremo Massif Protected Area, which covers most of the sites where these two species remain in the wild.

## Introduction

The flora of Madagascar exhibits remarkably high diversity and endemism, more than 80% of the approximately 14,000 known vascular plant species being unique to the country (Gautier & Goodman, 2003; Callmander, Schatz & Lowry, 2005; Goodman & Benstead, 2005; Buerki et al., 2011). Many plant families in Madagascar show high levels of diversity and endemism (including several fully endemic plant families: Asteropeiaceae, Barbeuiaceae, Physenaceae, Sarcolaenaceae and Sphaerosepalaceae), and it is widely acknowledged that a large proportion of Madagascar’s taxa are known to be facing significant threats to their continued survival (Myers et al., 2000; Ganzhorn et al., 2001; Kremen et al., 2008; Madagascan Ministry of Environment and Forests, 2014). The island faces some of the highest levels of habitat destruction in the world, from deforestation to soil erosion, and much of this destruction is driven by human activities (Ganzhorn, Rakotosamimanana & Hannah, 1997; Harper et al., 2007; Moat & Smith, 2007; Kremen et al., 2008; Allnutt et al., 2013). The need to conserve species in Madagascar—and more generally worldwide—is increasingly urgent, particularly given growing evidence of the likely impact of climate change on native plants in the future (Buerki et al., 2011; Foden et al., 2013; Madagascan Ministry of Environment and Forests, 2014; Brown et al., 2015; Scriven et al., 2015).

The Durban Accord and Action Plan, which emerged from the 2003 IUCN World Parks Congress (IUCN, 2003; Scales, 2014), committed Madagascar to conserve *in situ* all single-site species that are assessed using IUCN Red List categories and criteria as either Critically Endangered or Endangered (IUCN, 2005; Vié et al., 2008). The Durban Vision required Madagascar to triple its protected area coverage to protect 10% of its land surface (Norris, 2006; Scales, 2014). Progress is being made under the Système des Aires Protégées de Madagascar (SAPM) network to reach these commitments and approximately 4.3 million hectares of land, representing 102 sites across the island, have been formally identified as new protected areas (Système des Aires Protégées de Madagascar, 2016). Management plans are being developed for these newly designated protected areas, as well as studies of their constituent biodiversity and the conservation status of the species found in them.

How we utilise limited resources to conserve species requires decisions and trade-offs to be made. The relative importance of ecological versus genetic factors, under different scenarios, is an area of much research and discussion (Frankham, Ballou & Briscoe, 2002; Frankham, 2003). At the species-level, it is well documented that inbreeding and small (effective) population sizes will reduce the fitness of individuals and their future adaptability (Bijlsma, Bundgaard & Boerema, 2000; Brook et al., 2002; Blambert et al., 2016). Small populations are much more vulnerable to stochastic effects, more susceptible to extinction through disease, and more likely to be eradicated through climatic events such as flooding, and anthropogenic climate change, habitat fragmentation, and habitat destruction (Frankham, Ballou & Briscoe, 2002; Frankham, 2003; Kramer et al., 2008).

The palm family (Arecaceae) in Madagascar comprises 204 species in 17 genera (Govaerts et al., 2016), 98% of which are endemic to the country. A recent study showed that 83% of the endemic palm species in Madagascar are threatened with extinction (Rakotoarinivo et al., 2014), compared with a global trend of just over 20% of all plant species being threatened with extinction, based on a sampled approach across all vascular plants (Brummitt & Bachman, 2010; Brummitt et al., 2015), and 50% of Madagascan non-palm vascular plants (IUCN, 2017). Rakotoarinivo et al. (2014) highlighted the conservation importance of palms in Madagascar and the urgent need to protect remaining populations. Many of these palm species have highly restricted and often fragmented wild populations, and more than half of the endemic species are known from fewer than 100 mature adult individuals.

Many studies of extinction risk and genetic factors in palms have been undertaken in recent years, including several in Madagascar (Shapcott et al., 2007; Shapcott et al., 2012; Shapcott, Quinn & Rakotoarinivo, 2014). A study of *Beccariophoenix madagascariensis* by Shapcott et al. (2007) found that this Vulnerable species showed considerable genetic diversity within populations, and that geographically and ecologically separated populations were clearly genetically distinct. Another study by Shapcott et al. (2012), and see also Shapcott, Quinn & Rakotoarinivo (2014), showed that although the Critically Endangered *Voanioala gerardii* has extremely small populations in the wild, the species contains an unexpectedly high level of genetic diversity, contrasting with the known populations of the Endangered *Lemurophoenix halleuxii* which exhibit a low level of genetic variation.

Population-level studies such as these often conclude with recommendations for the future conservation of their study species and similar taxa, highlighting the need for *in situ* protection of sites, the collection of seeds and plants for *ex situ* seedbanks, and the cultivation of species in botanic gardens and field gene banks. Other studies consider how representative *ex situ* collections such as botanic gardens and seed banks are of species’ genetic diversity (Guerrant et al., 2014; Guja et al., 2015), and the integration of genetic theory and information in conservation management planning (IUCN/SSC, 2008; IUCN/SSC, 2013; Wang et al., 2015).

**Figure 1:**
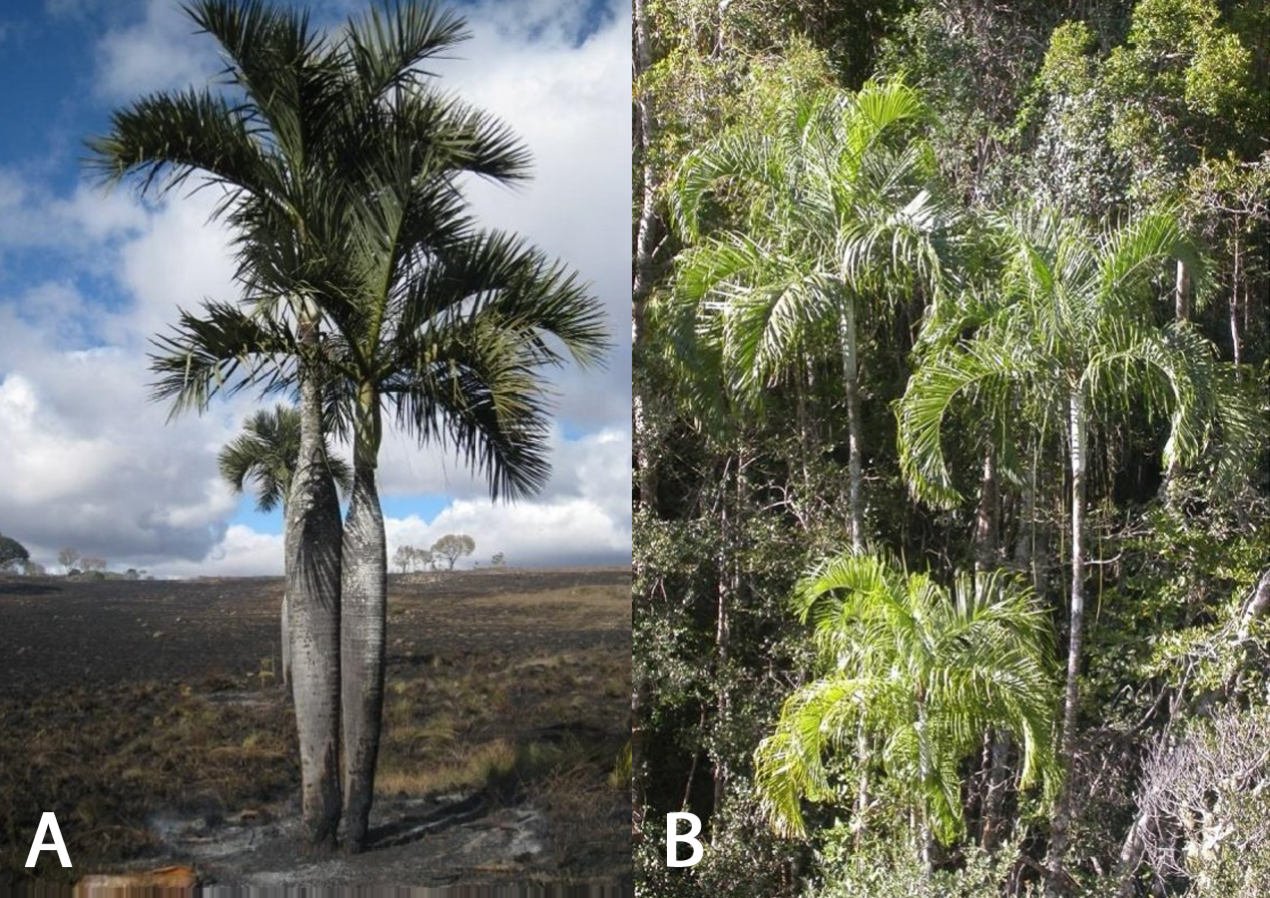
(A) *Dypsis decipiens* (IUCN: Endangered), (B) *Dypsis ambositrae* (IUCN: Critically Endangered), palms found in the central highlands of Madagascar. Photo credits: (a) L Gardiner, (b) M Rakotoarinivo.

The objectives of this study were to determine the utility of assessing the genetic diversity and structure of two endemic threatened palm species found in the Itremo Massif in the Central Highlands of Madagascar. The study assesses the genetic diversity of *Dypsis decipiens* (Fig. 1A) and *D. ambositrae* (Fig. 1B), and the degree of gene flow and inbreeding among populations in the wild. Amplified Fragment Length Polymorphism (AFLP) markers are widely used in population genetics and conservation, and their use does not require the time- and resource-intensive initial steps required for genome-specific microsatellite SSRs. Given limited resources for conservation of palms in Madagascar, this approach was used to inform appropriate *in situ* and *ex situ* conservation management of these two species in the future. The results of this work should inform future conservation work on palms, and other threatened taxa, both in Madagascar and in other countries.

## Materials and Methods

### Study sites and target species

The Itremo Massif, officially designated in 2015 as a protected area (Madagascan Ministry of Environment, Ecology, Sea and Forests, 2015) and part of the SAPM network created out of the Durban Vision, is located in the Central Highlands of Madagascar, to the west of Ambositra in the Amoron’i Mania region. The massif is characterised by an outcrop of metamorphic rocks dominated by quartzite and schist over a distance of approximately 120 km, lying north to south, interrupted frequently by open plains (Cox, Armstrong & Ashwal, 1998). Ranging in elevation between 1,500 and 2,100 m above sea level, the geomorphology of the whole area is generally abrupt and its bioclimatic type is warm subhumid, characterised by ca 1,500 mm of annual precipitation, a mean average temperature of 25 °C, and up to seven dry months (Cornet, 1974). Given these physical settings, Itremo’s biodiversity is recognised as particularly unique with a flora including many narrow endemics recorded from several distinct vegetation types, dominated by xerophytic rocky outcrops, grassland, Tapia woodland and gallery forest (Moat & Smith, 2007).

Four palm species have been recorded from this area: *Dypsis ambositrae* Beentje, *D. baronii* (Becc.) Beentje & J Dransf., *D. decipiens* (Becc.) Beentje & J Dransf.*,* and *Ravenea madagascariensis* Becc. Two of these species, *D. ambositrae* and *D. decipiens*, have been assessed as Critically Endangered and Endangered respectively, using IUCN Red List categories and criteria (version 3.1) (Vié et al., 2008; Rakotoarinivo & Dransfield, 2012a; Rakotoarinivo & Dransfield, 2012b; Rakotoarinivo et al., 2014). *Dypsis baronii* and *R. madagascariensis* were assessed using the same categories and criteria and found to be of Least Concern, so were not included in this study (Rakotoarinivo & Dransfield, 2012c; Rakotoarinivo & Dransfield, 2012d).

**Figure 2:**
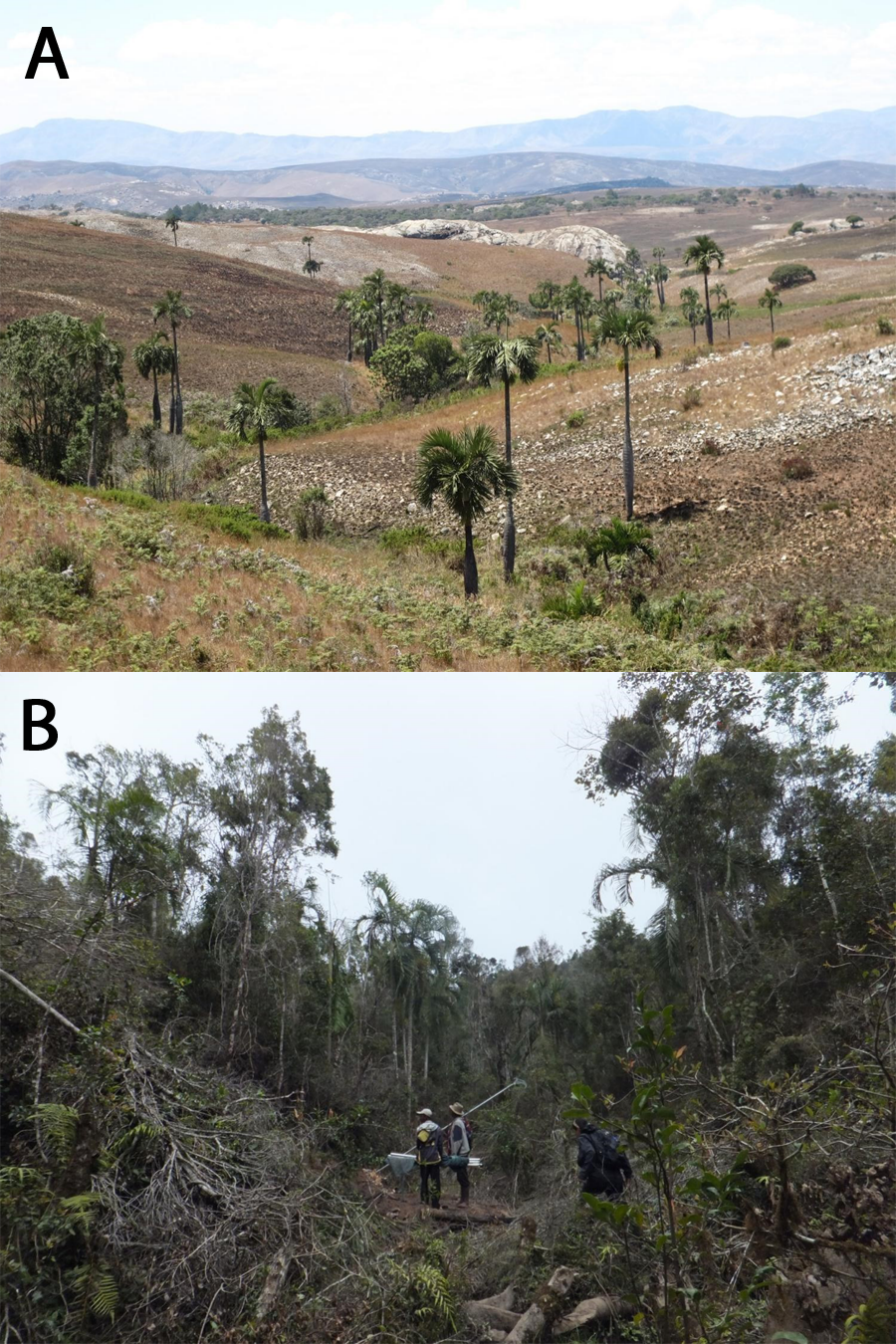
(A) *Dypsis decipiens* grows in large but widely spaced stands across the Itremo Massif of the Madagascan Central Highlands, a region vulnerable to frequent and extensive grassland fires. (B) *Dypsis ambositrae* grows in small populations in riverine and gallery forest in Madagascar, vulnerable to deforestation and charcoal production. Photo credits: L Gardiner.

*Dypsis decipiens* is found across the Central High Plateau of Madagascar between Andilamena and Fianarantsoa. In the Itremo Massif Protected Area, the species occurs in large stands of hundreds of individuals, along riverine valleys and hillsides, in open grasslands, and it has been estimated that at least 2,700 individuals may grow here (Fig. 2A) (Rakotoarinivo & Rajaovelona, 2013). In contrast, *D. ambositrae* is restricted to much smaller numbers. Fewer than 50 mature individuals were known in the wild at the start of this work, all restricted to gallery forest in the Itremo and Ambositra areas (Fig. 2B) (Rakotoarinivo & Rajaovelona, 2013).

### Sampling strategy

**Figure 3:**
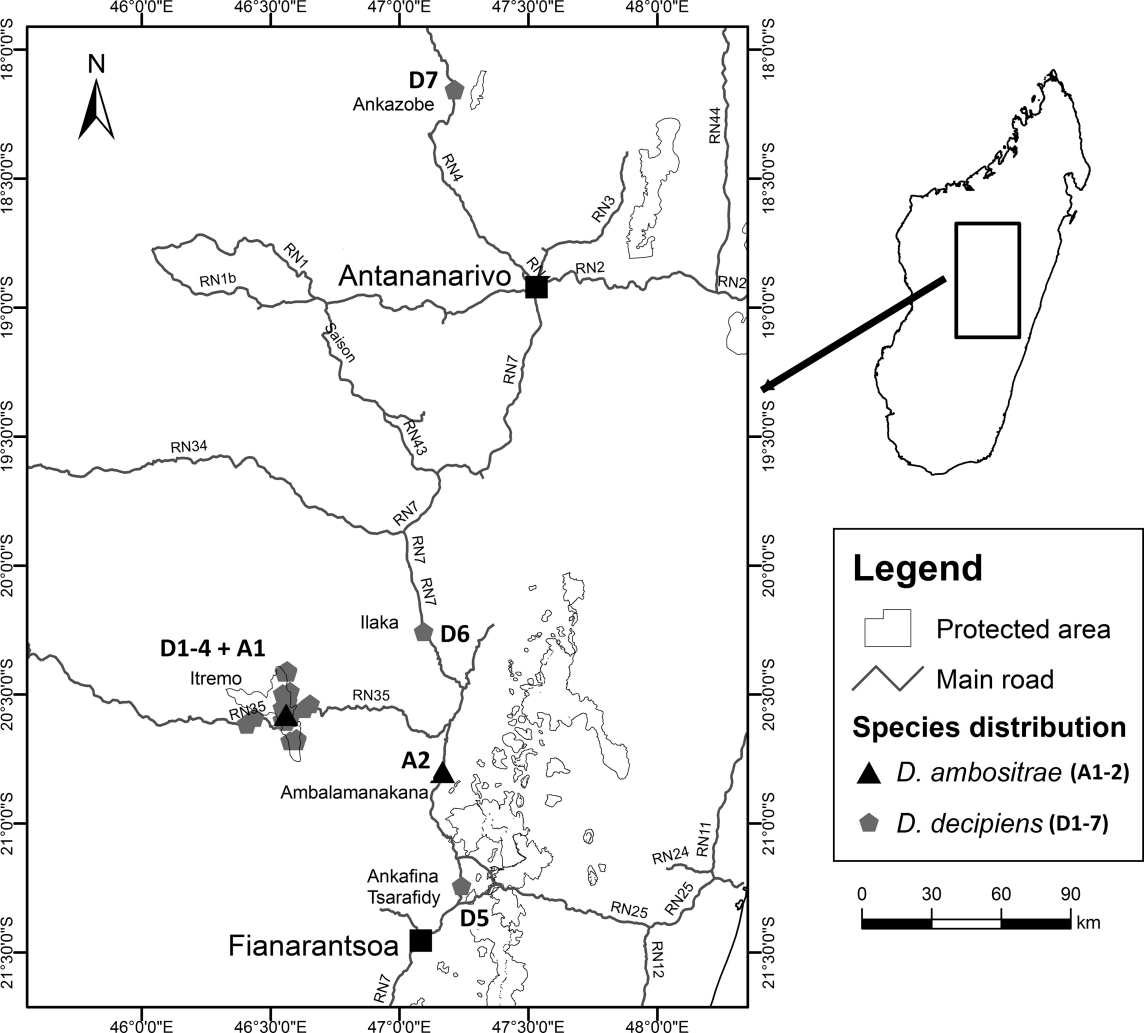
Locations of collection sites for *Dypsis ambositrae* and *D. decipiens*.

**Figure 4:**
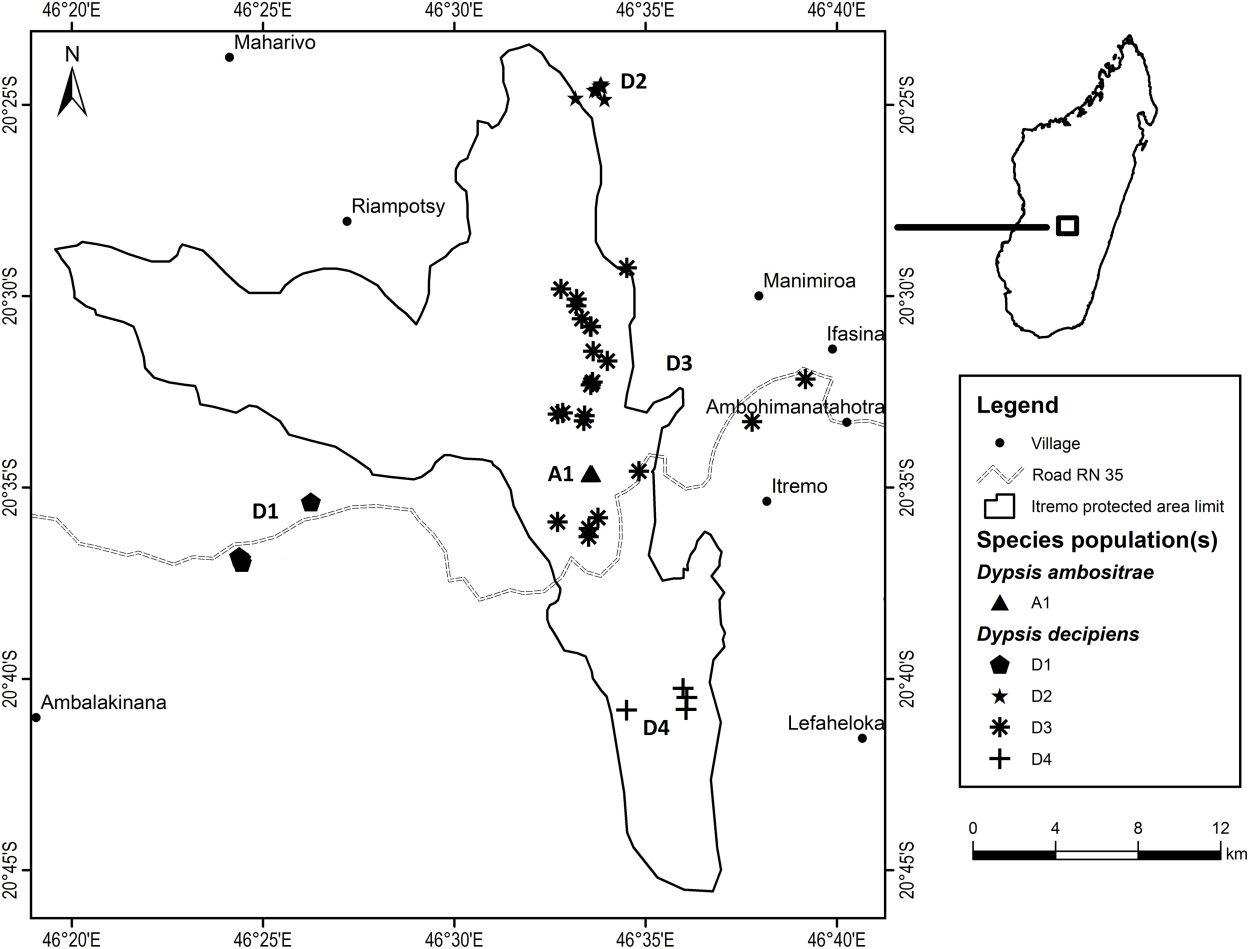
Detailed map of *Dypsis ambositrae* and *D. decipiens* collection sites within the Itremo Massif region, Madagascar.

Fieldwork took place between July 2012 and March 2013 as part of a Conservation Leadership Project managed by Kew Madagascar Conservation Centre, under research permits issued by the Madagascan Ministry of Environment and Forests (permit number: 151/12/MEF/SG/DGF/DCB/SAP/SCB, issued to Mijoro Rakotoarinivo). Samples of leaf tissue were dried in silica gel to preserve their DNA. The majority of specimens were collected from the Itremo Massif region, where the population sizes of the two species are largest; other known sites for the two species visited and sampled were Ankazobe, Ilaka, Ambalamanakana, and Ankafina Tsarafidy (Fig. 3). There are some unconfirmed reports of small numbers of *D. decipiens* also being found east of Antananarivo, but these sites were not sampled during this study. For the large populations of *D. decipiens* in the Itremo Massif (Fig. 4), a 1 km^2^ sampling grid was overlain on satellite imagery of the region. Only this one large species of palm is found in the open grassland of this region, so individual palms could be mapped accurately and, using the grid, leaf material could be collected from individuals from each grid cell in the field.

### DNA extraction

DNA was extracted from the silica-dried leaf samples using a modified CTAB method (Doyle & Doyle, 1990)—washing the DNA pellet with 70% ethanol, drying it at 37 °C, and resuspending in TE buffer (20 mmol Tris-HCl, 0.1 mmol EDTA) (Rivers et al., 2011; Devey et al., 2013)—and the DNA concentration was quantified using a Nanodrop spectrophotometer.

### AFLP methods

AFLP methods followed those described by Vos et al. (1995). *Eco*RI and *Mse*I restriction enzymes were used to digest each DNA sample, and double stranded *Eco*RI and *Mse*I adaptors were ligated onto the ends of the digested fragments. A primer trial was conducted using 12 primer pairs, to select three combinations that would provide a level of variation appropriate for this study. Primer combinations *Mse*I-CTC + *Eco*RI-AC, *Mse*I-CTG + *Eco*RI-AAG, and *Mse*I-CAC + *Eco*RI-AAC, were used in the analysis, the selective *Eco*RI primer being labelled with a fluorescent dye, and a 250 ROX size ladder being used. Resulting fragments were analysed using an ABI 3730 Genetic Analyser. GeneMapper (Applied Biosystems, 2005) was used to automatically score the presence and absence of fragments, ranging between 40 bp and 400 bp, in a binary matrix for all samples. Scoring of every electropherogram was manually checked to confirm the presence or absence of additional fragments that the programme was unable to unambiguously score automatically.

DNA samples collected in the field which did not produce consistent, scoreable AFLP bands, were excluded from subsequent scoring and analyses (see ‘Discussion’).

### AFLP data analysis

Descriptive statistics for the *D. ambositrae* and *D.decipiens* datasets were calculated using AFLPsurv (Vekemans, 2002), using values of the within-population inbreeding coefficient *F*_IS_ calculated in I4A (Chybicki, Oleksa & Burczyk, 2011) and GenAlEx v.6.3.1 (Peakall & Smouse, 2006; Peakall & Smouse, 2012).

Relationships among populations of both species were investigated by calculating Nei’s genetic distances to generate genetic distance matrices, visualising the relationships between populations and individuals using Principal Coordinates Analysis (PCoA) (Gower, 1971) in MVSP (Kovach, 2013) and GenAlEx (Peakall & Smouse, 2006; Peakall & Smouse, 2012) to assess the distinctiveness of populations.

The significance of the partitioning of AFLP variation among populations was tested using Analysis of Molecular Variance (AMOVA) in GenAlEx using 999 permutations, and a Mantel test was implemented in GenAlEx to test for Isolation by Distance (IBD), comparing genetic and geographic distance matrices for both species.

## Results

### AFLP analysis

AFLP profiles with data from all three primer combinations were produced and analysed for 20 *D. ambositrae* and 50 *D. decipiens* individuals. Descriptive statistics of the results for both species are shown in Table 1.

**Table 1:** Descriptive statistics of species datasets for both *Dypsis ambositrae* and *D. decipiens*.

Population	*N*	*F*_ST_	*F*_IS_	*H*_*j*_	*H*_*w*_	*H*_*t*_	*H*_*b*_	Nei’s	Bands	% P	pB	I
*D. ambositrae*												
A1	8			0.232					193	40.4	21	0.200
A2	12			0.233					199	52.6	27	0.261
**Total/mean**	**20**	**0.095**	**0.117**	**0.232**	**0.233**	**0.257**	**0.024**	**0.032**	**196**	**46.5**	**24**	**0.230**
*D. decipiens*												
D1	5			0.251					258	41.9	1	0.218
D2	9			0.221					284	49.7	2	0.236
D3	25			0.202					319	63.0	10	0.265
D4	4			0.242					245	34.6	1	0.185
D5	3			0.222					224	23.2	0	0.129
D6	2			0.207					196	12.2	0	0.074
D7	2			0.332					250	31.9	16	0.193
**Total/mean**	**50**	**0.081**	**0.270**	**0.239**	**0.239**	**0.260**	**0.021**	**0.028**	**254**	**36.6**	**4**	**0.186**

**Notes.**

*N* number of individuals analysed *F*_ST_ mean genetic differentiation among populations *F*_IS_ mean within-population inbreeding coefficient *H*_*j*_ expected heterozygosity *H*_*w*_ mean within-population expected heterozygosity *H*_*t*_ total genetic diversity *H*_*b*_ mean genetic diversity among populations Nei’s average Nei’s genetic distance across all populations bands number of different bands %P percentage of polymorphic loci pB number of private bands I Shannon’s diversity index

#### Genetic diversity

Altogether a total of 370 loci were scored from 50 *D. decipiens* individuals, from seven geographically distinct populations. A total of 135 loci were produced using the primers *Mse*I-CTC + *Eco*RI-AC, 113 loci from *Mse*I-CTG + *Eco*RI-AAG, and 122 loci from *Mse*I-CAC + *Eco*RI-AAC.

A total of 230 loci were scored from 20 *D. ambositrae* individuals, from 2 geographically distinct populations. 87 loci were produced using the primers *Mse*I-CTC + *Eco*RI-AC, 67 loci from *Mse*I-CTG + *Eco*RI-AAG, and 76 loci from *Mse*I-CAC + *Eco*RI-AAC.

Descriptive statistics for both species can be seen in Table 1.

The lowest levels of diversity are seen in the samples from the smaller size subpopulations of *D. decipiens*, with the higher levels in populations D2 and D3, where more samples were collected.

#### Genetic structure

**Figure 5:**
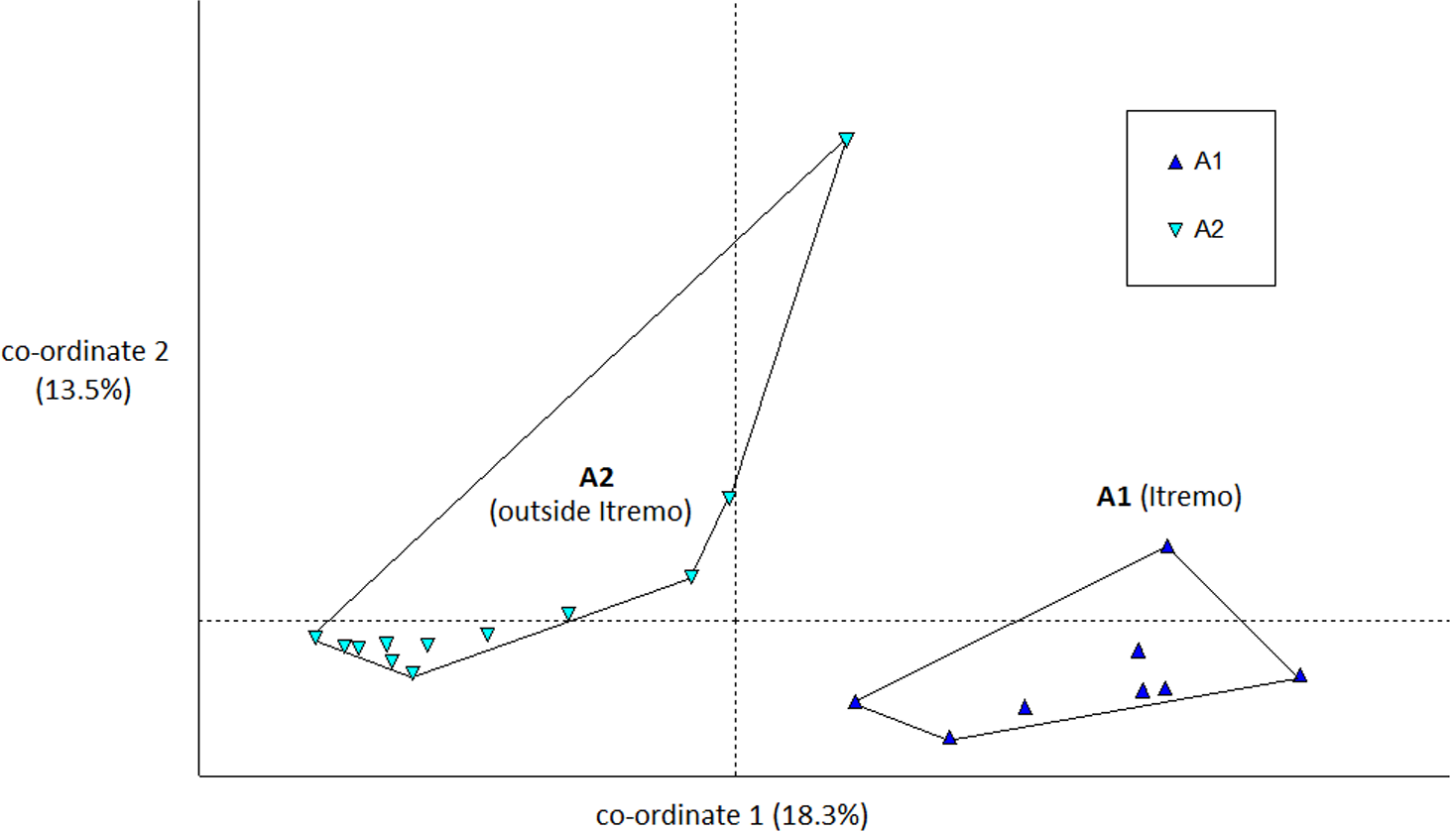
PCoA plot of AFLP variation in *Dypsis ambositrae* individuals sampled from two geographic locations in Madagascar. The clustering of samples from each populations is indicated using convex hulls.

**Figure 6:**
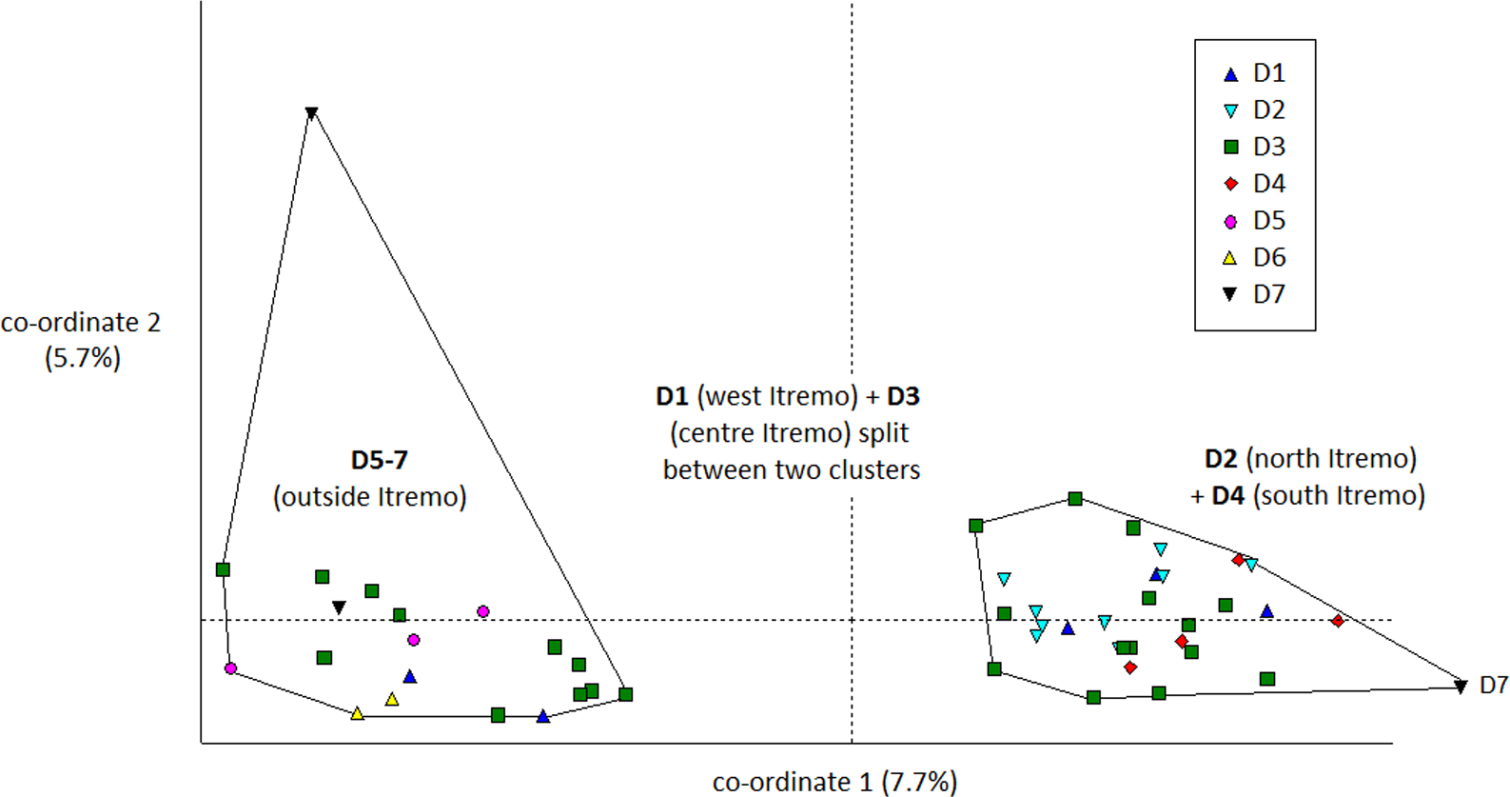
PCoA plot of AFLP variation in *Dypsis decipiens* individuals sampled from seven geographic locations in Madagascar. The clustering of samples from each populations is indicated.

The PCoA results using Euclidean genetic distances between individuals show that there is some clear separation of the genetic variation in the two species into clusters. The two populations of *D. ambositrae* separate into two separate clusters using the first two PCoA axes (Fig. 5). The clusters visualised for *D. decipiens* using PCoA axes 1 and 2 (Fig. 6) do not separate the seven populations into seven separate groups, but there is some pattern—the two clusters seen comprise populations D2 and D4 in one cluster, and D5, D6 and D7 in the other. Individuals from populations D1 and D3 are divided between the two groups. The proportion of variation represented by each of the first two axes is shown in Figs. 5 and 6 (*D. ambositrae*: 18.3% and 13.5% respectively, *D. decipiens*: 7.7% and 5.7%). The third axis for each species (not shown) adds an additional 9.5% and 4.3% of the variation but reveals no more clustering of the individual points. Oddly, for both species, there is a single outlying individual. The outlying individual for *D. ambositrae* is a sample collected from the A2 population, outside the Itremo protected area. The outlying individual of *D. decipiens* is one of just two individuals collected from the D7 population north of Antananarivo (the other individual is found within the main D5-7 cluster of individuals in the PCoA).

The AMOVA test (Table 2) shows that in both species the majority of the genetic diversity is evident within populations rather than among them (79% and 93% of the variation for *D. ambositrae* and *D. decipiens* respectively); thus, more of the genetic variation in *D. ambositrae* is related to among population variation than in *D. decipiens* (21% compared with 7%), though it is recognised that the number of subpopulations studied for each species differ substantially. The pairwise genetic distances among sample sites (*ϕ*PT) was much higher for *D ambositrae* (0.211) compared with those for *D. decipiens* (0.074).

**Table 2:** Results of AMOVA conducted on variation among and within populations of *Dypsis ambositrae* and *D. decipiens*. The data include the percentage of variation explained by the different hierarchical levels, and Mantel test results comparing linear genetic distance and geographic distance matrices between individuals of each species.

		Analysis of Molecular Variance (AMOVA)							Mantel	
		df	SS	MS	Est. var.	%	*ϕ*PT	P	Rxy	P
*D. ambositrae*	Among pops	1	61.400	61.400	4.600	21				
	Within pops	18	310.250	17.236	17.236	79				
Total		19	371.650		21.837		0.211	0.001^**^	0.344	0.010^*^
*D. decipiens*	Among pops	6	305.866	50.978	2.778	7				
	Within pops	43	1500.874	34.904	34.904	93				
Total		49	1806.740		37.682		0.074	0.001^**^	0.562	0.010^*^

**Notes.**

Tests of significance

* *P* < 0.05.

** *P* < 0.01, based on 999 permutations.

#### Geographic structure

The Mantel test shows that for both species, there is evidence of Isolation by Distance; the genetic and geographic distance matrices are statistically significantly related to each other (*P* < 0.001). As visualised in the PCoA results (Fig. 5), the two geographically separate populations of *D. ambositrae* are genetically distinct from each other, and the Mantel test results confirm the statistically significant geographical structure of the genetic diversity present in this species. Similarly, the Mantel test confirms the presence of statistically significant geographical structure in the genetic diversity of the *D. decipiens* individuals, among the populations D5, D6 and D7, and between populations D2 and D4 (as visualised in Fig. 6).

## Discussion

During the course of this work, much information was collected on the distributions of the two palm species studied here for the first time. Both species were confirmed as being centred on the Itremo Massif region in the Central Highlands of Madagascar.

We now believe that *D. ambositrae* has a very small known global population of no more than 100 mature individuals in the wild, split between two sites. One population (A1) is found within the Itremo Protected Area, in a relatively undisturbed area of humid gallery forest. The second population (A2) occurs close to a major road (Route National 7), in a fragment of forest that has been heavily degraded in recent years (although local people have made efforts to protect it). Degradation has been particularly due to the decline in habitat quality, through the extraction of large (non-palm) trees for *in situ* charcoal production, and the felling of the palms to facilitate access to these trees.

*Dypsis decipiens* is found across a much larger area than previously thought; we now estimate that 2,500–3,000 individuals grow across the open grassland of the Itremo Massif region, and are grouped into the subpopulations shown in Figs. 3 and 4. A large central population (D3) is flanked by smaller clusters of individuals to the north and south (D2 and D4, respectively), and by a small population to the west (D1) along the road crossing the country West–East, through the Central Highlands. The species is also now known to occupy at least three other sites, all north of Itremo and near major roads. Populations D5 and D6 (near RN 7) are found between Antsirabe and Fianarantsoa, and D7 (near RN 4) is north of the capital Antananarivo.

It is worth noting that not all of the silica-dried samples collected across the region yielded DNA of adequate quality for analysis; this is likely to have been due to problems with drying some samples sufficiently quickly in the field. Other researchers have anecdotally reported problems with extracting DNA from palm leaf material, possibly due to the presence of mucilage and secondary metabolites that are thought to either interfere with the extraction process or lead to the degradation the DNA after collection in the field.

### Genetic diversity

This study has shown that overall genetic diversity and levels of heterozygosity are fairly low in both of the study species. Comparatively higher levels of genetic diversity are known to enhance species’ resilience and persistence in the wild, so these results raise concerns about the future ability of these two palms to survive in the future in the face of climate change and encroaching human-induced habitat destruction. Interestingly, the more widespread species *D. decipiens* actually has lower genetic diversity than *D. ambositrae*, the species with the dramatically smaller and geographically restricted population.

The low proportion of polymorphic loci revealed in the two palm species (46.5% and 36.6%) is comparable with the low proportion found in a similar AFLP study of Madagascan legume tree species, where an average of 40% of loci were found to be polymorphic, and the more threatened species were found to have fewer polymorphic loci than the less threatened taxa (Rivers et al., 2011). Low genetic diversity has also been reported in several threatened palms elsewhere in the world (Dowe, Benzie & Ballment, 1997; Shapcott, 1998; González-Pérez, Caujape-Castells & Sosa, 2004), but in other studies substantially higher genetic diversity has been revealed (Shapcott, 1999; Henderson, Billotte & Pintaud, 2006; Shapcott et al., 2007).

Compared with another study of Madagascan palm population genetics using AFLPs, the study of *Beccariophoenix madagascarienis* (Shapcott et al., 2007), the measures of genetic diversity revealed in here seem to be low. For *B. madagascariensis,* mean genetic differentiation among populations (*F*_ST_) is much higher (at 0.599, versus 0.095 and 0.081 respectively for *D. ambositrae* and *D. decipiens*), and similarly, the expected heterozygosity (*H*_e_) is 0.595–0.691 for *B. madagascariensis*, versus 0.233 and 0.239 for *D. ambositrae* and *D. decipiens.* In the Madagascan legume AFLP study of Rivers et al. (2011), species considered to be of higher threat status were also shown to have lower genetic diversity indices, including the Shannon Diversity Index, as seen in this study for both *D. ambositrae* and *D. decipiens*). A study of two Madagascan palms, *Voanioala gerardii* and *Lemurophoenix halleuxii* (Shapcott et al., 2012), showed contrasting patterns—demonstrating high genetic diversity in the former but low genetic diversity in the latter, but used microsatellites rather than AFLPs.

### Genetic and geographic structure

There is clear evidence for a degree of genetic and geographic clustering in both species. Clustering is more clearly defined in *D. ambositrae*, with two genetically distinct populations, which correspond to the two geographically distinct populations (A1 and A2), being revealed by the AFLP analysis. In *D. decipiens*, there seems to be some genetic structuring dividing the sampled individuals into two main groups, but these do not correspond completely with the geographically distinct populations. There could be said to be a core Itremo genetic group, comprising individuals from D1 (west Itremo), D2 (north Itremo), D3 (central Itremo) and D4 (south Itremo), and a separate genetic group that follows the road network through Itremo from the west to the centre, and northward along routes RN 4 and 7. The distribution of this latter group could reflect the anthropogenic movement of the species along the road system.

### Conservation recommendations

The population sizes and genetic and geographic groupings revealed in this study can be used to inform conservation decision making for the two species studied, and already are being taken into account in current conservation efforts.

The overall genetic diversity represented in both species is so low that extensive, time- and funding-consuming efforts to try to conserve representatives from across their entire distributions (e.g., in comprehensive seed collections) would not be the best use of available resources, as most of the limited genetic diversity would be captured by relatively few individuals. Although we might predict threatened species to represent low genetic diversity compared with less threatened species, and the results of this study are congruent with this theory, the results of these other studies suggest that this rule does not always apply and cannot be assumed for all species. Further study of biological and ecological traits of threatened taxa, including life history traits, population distributions and trends, geographic and habitat preferences, may help to interpret these conclusions further.

The results of this study suggest that it should be a priority to collect seed from both *D. ambositae* populations in order to capture the maximum genetic diversity. This should result in *ex situ* seed and living collections being that which represent as many unique alleles as possible, improving the genetic basis upon which future re-introduction and supplementation work would be based. *In situ* efforts should aim to protect both of the remaining populations from future degradation—although one population is protected within Itremo NPA, the population near route RN7 is highly threatened.

The situation with *D. decipiens* is perhaps less urgent than is the case with *D. ambositrae,* which has a much larger total population size. Moreover, most of the plants are found within the Itremo NPA, a legally designated site managed for species and habitat conservation objectives by RBG Kew with strong community engagement and development work. Interestingly, this study has shown that the genetic groupings of the species are limited to just two main clusters, and these do not correspond well with the geographic sites from across the full distribution of the species, from which the plants were sampled. Resource-intense efforts could in theory be made to collect *D. decipiens* seed from across its full distribution in order to capture as much genetic diversity as possible, but the results of this study show that increased sampling effort may well result in limited gains in terms of genetic diversity represented in the collected material.

The small populations of *D. decipiens* occurring outside the Itremo Massif area do not represent significant genetic diversity beyond that represented in the main populations within Itremo NPA, and so are less important for conservation either *in situ* or *ex situ*. We hypothesis that these small populations, reliably found near main roads, may be the result of humans deliberately moving these aesthetically attractive plants.

Since most individuals of *D. decipiens* grow within the Itremo NPA, and therefore are conserved *in situ*, our main recommendation would be ensure that the species is well represented in *ex situ* collections, ideally by progeny from a range of mother plants. As with other horticulturally desirable species, *D. decipiens* is widely grown around the world. However, in the case of species such as this, confined in the wild to remote islands like Madagascar, cultivated plants are likely to be the result of just a small number of original exported seed collections–and therefore represent a very limited gene pool from the species in the wild. This certainly applies to the Critically Endangered palm *Tahina spectabilis*, although represented in many *ex situ* collections around the world, virtually all plants are progeny of a single seed collection taken from just one individual in the wild (J Dransfield, pers. comm., 2016; Gardiner et al., in press). In the case of *D. decipiens*, collections of seed and/or seedlings from a greater number of individuals from within the Itremo NPA would likely improve the genetic diversity represented in *ex situ* collections. The lack of clear genetic groupings each directly corresponding to particular geographic populations means that seed collections cannot (and need not) be gathered from across the entire geographic distribution of the species, and less structured and more opportunistic collecting may well result in the collection of as much genetic diversity as a more systematic, geographically even but labour-intensive approach.

## Conclusions

The utility of employing population genetic techniques in conservation situations where population sizes are extremely small and/or threatened may, in some cases, be limited when resources (including time) are severely restricted. In the situation presented in this study, the amount of information obtained from assessing the genetic diversity and structure of the two study species proved to be relatively limited. It is also unclear how far one can extrapolate from these results to other palm species in Madagascar, or more generally to other island species.

The results of this work do usefully support current priorities of conservation organisations to concentrate specific resources on conserving otherwise unprotected small populations and fragmented sites (such as the *D. ambositrae* population located outside the Itremo Protected Area), with less focus on additional conservation measures for species already enclosed within protected areas and/or with larger and more continuous populations (as for *D. decipiens*). *In situ* species conservation should continue to be the priority, but in terms of *ex situ* conservation, the priority when resources are limited should be to sample small populations across their distribution, thereby capturing as much genetic diversity in collections as possible. Less effort need be expended on sampling extensively across larger, and less fragmented, populations.

##  Supplemental Information

10.7717/peerj.3248/supp-1 Supplemental Information 1 *Dypsis ambositrae* AFLP data AFLP coded data for 20 *Dypsis ambositrae* samples, 3 primer combinations, 230 loci.

10.7717/peerj.3248/supp-2 Supplemental Information 2 *Dypsis decipiens* AFLP data AFLP coded data for 50 *Dypsis decipiens* samples, 3 primer combinations, 370 loci.

## Additional Information and Declarations

### Field Study Permissions

The following information was supplied relating to field study approvals (i.e., approving body and any reference numbers): The research permit (number: 151/12/MEF/SG/DGF/DCB/SAP/SCB, issued to Mijoro Rakotoarinivo as the field lead) for the collection of material used in this study was issued by the Madagascan Ministry of Environment and Forests.

### Data Availability

The following information was supplied regarding data availability: The raw data has been supplied as Supplemental Dataset.
